# Construction Notes

**Published:** 1904-10-15

**Authors:** 


### CONSTRUCTION NOTES.

A new operating theatre has been opened at the Ulverston Cottage Hospital.

A new nurses' home has been opened at the Norwich Workhouse Infirmary.

The new administrative buildings of the epileptic colony at Chalfont St. Peters will be opened on October 20th.

The Acton Cottage Hospital has been enlarged at the cost of £1,000, towards which Mr. Passmore Edwards contributed £500.

An anonymous donor has given £1,000 to the Cardiff Infirmary to endow a cot, where further accommodation is much needed.

The late Mr. J. G. Hill, of Sunderland, has bequeathed £500 to the Sunderland Infirmary and £250 to the North Durham Eye Infirmary.

Mr. Frederick Ransome, of Ipswich, has endowed a bed at the Ipswich Hospital, to be called the Frederick and Amy Ransome Memorial Bed.

A new out-patients' department has been added to the Ipswich Hospital. It is a memorial to the late Dr. Bartlett, formerly a member of the honorary staff of the hospital, and has been erected by his son. It is modelled after the out-patients' department at the London Hospital.

The former Prince Alfred Hospital, Sydney, is now The Royal Prince Alfred Hospital, by permission of the King. The new Queen Victoria Memorial Pavilions are progressing towards completion. The chairman, Professor Anderson Stuart, paid a recent visit to many hospitals in Europe, and on his return, in the light of his experience, expressed himself very satisfied with the additions to the hospital now in progress. The proposed arrangements for the operating theatre, he thinks, bear comparison with the best he has seen elsewhere.

At the present time Hambledon sends its isolation cases to Farnham, but in 1907 the arrangement is to cease, and it is proposed for convenience sake that Hambledon should have an isolation hospital. A site has been selected and preliminary plans prepared, but at the special request of the ratepayers, the consideration of the scheme is postponed for a year on the plea of "bad times." The estimated cost of £7,000 is considered too high, and the architect is to be requested to prepare plans on a cheaper basis. It is to be hoped that before Hambledon spends its money that it will ascertain what it really needs, and then be prepared to see that they are efficiently provided for. District councillors cannot be expected to possess the medical and architectural knowledge necessary to enable them to instruct an architect to reduce the cost of a building by some thousands of pounds, without seriously endangering the efficiency and utility. There is plenty of time fortunately in which inquiries can be made into the cost of providing reasonable isolation accommodation for similar communities.

